# Connecting self-esteem to problematic AI chatbot use: the multiple mediating roles of positive and negative psychological states

**DOI:** 10.3389/fpsyg.2025.1453072

**Published:** 2025-03-24

**Authors:** Ruiqi Yao, Guijie Qi, Dongfang Sheng, Hua Sun, Jiacheng Zhang

**Affiliations:** ^1^School of Management, Shandong University, Jinan, China; ^2^School of Journalism and Communication, Tsinghua University, Beijing, China

**Keywords:** problematic technology use, AI chatbots, compensatory internet use theory, flow experience, self-esteem

## Abstract

The emergence of AI chatbot products has ushered in a new era of human-AI interaction, yet scholars and practitioners have expressed concerns about their use due to potential addictive and adverse effects. Currently, the understanding of problematic AI chatbot use (PACU) remains incomplete and inconclusive. Despite previous findings that indicate negative outcomes associated with the use of AI products, limited studies have explored the underlying factors that drive the complex process leading to the formation of PACU. Furthermore, while existing literature highlights how personal traits influences problematic IT use via evoked psychological states, it largely overlooks that the positive psychological experience may also have a potential influence on problematic outcomes. Incorporating flow experience into the compensatory internet use theory, this study presents a multiple mediation model to investigate how social anxiety, escapism, and AI chatbot flow influence the relationship between self-esteem and PACU. We examine the model using Partial Least Squares Structural Equation Modeling (PLS-SEM) with cross-sectional data collected from 563 online users who have engaged with AI chatbots. Our findings indicate that users with low self-esteem are more likely to conduct problematic behavior when using AI chatbots. This relationship can be mediated by social anxiety, escapism and AI chatbot flow. This study sheds light on how self-esteem negatively affects PACU, unraveling the underlying psychological processes experienced by users with low self-esteem in their interactions with AI chatbots. Also, we provide practical insights for online users and practitioners to mitigate the potential negative impacts of AI product usage.

## 1 Introduction

Artificial intelligence (AI), which is increasingly relied upon as a catalyst for envisioning a brighter future, has witnessed remarkable advancements in recent years, particularly in the domain of natural language processing (NLP) (Adamopoulou and Moussiades, 2020). The use of AI chatbots has shown a worldwide prairie ablaze. For instance, Replika has over 10 million users as of February 2023 and is ranked as the #1 social networking app on the App Store and Google Play. Traditional chatbots were primarily rule-based, with their interactions pre-programmed according to specific rules. In contrast, the new generation of AI chatbots significantly extends the capabilities of their predecessors by leveraging large-scale language models endowed with powerful text generation abilities (Malik et al., 2023). These advanced chatbots can communicate with users, grasp real-time intents, and offer various content they need due to the ability to self-learn and even utilize search engines (Zhai and Wibowo, 2022).

AI chatbots have become increasingly popular because they greatly improve people’s quality of life. For humans who live under high pressure and fast pace, chatbots provide fun and convenience. However, the over-reliance on AI chatbots has aroused people’s concerns and worries about some potential side effects of using them. The average session duration of AI chatbots is increasing across the board. With a widespread outage of ChatGPT on March 20th that made the AI chatbot inaccessible to its users, heavy users started to express their anxiety on social media. Some of them wrote on Instagram that they ‘felt like a child who lost their mom in the grocery store when found it cannot function’. Researchers have realized the adverse effects of AI chatbots which prevent people from further utilizing them (Coombs et al., 2021). Some empirical evidences have already been provided that AI chatbots generally are highly addictive thus leading to a potentially excessive and maladaptive chatbot use behavior, which we can term problematic AI chatbot use (PACU) (Xie and Pentina, 2022; Pentina et al., 2023). A large number of users seek emotional support from chatbots, who can understand and influence their innate feelings, and their original psychological states are changed by AI chatbots when users perceive that AI chatbots care about them (Xu et al., 2017; Adamopoulou and Moussiades, 2020; Kautish and Khare, 2022). This produces a psychological connection that people may get attached to AI chatbots and develop PACU. PACU is just like taking mood-altering or hallucinogenic drugs in order to produce the happy illusion instead of actually becoming happy which can impair people’s innate mental capacities. It represents the loss of self-reliance in solving problems, and even isolation from the real world (Hu et al., 2023). Individuals who fall into PACU may experience increased levels of anxiety and depression due to their excessive reliance on AI chatbots for emotional support. This overreliance can also impair their real-life social interactions, leading to isolation and strained relationships. Therefore, it is better to batten down the hatches and reveal the internal mechanism of PACU from an empirical perspective.

Psychological researchers have focused on human-chatbot interaction, user satisfaction and trust, arousing discussions in the fields of information science, healthcare, marketing, e-commerce, and education (Li and Sung, 2021; Jiang et al., 2022; Zhai and Wibowo, 2022; Konya-Baumbach et al., 2023). In the prior literature, the effects of personal traits on problematic IT use through psychological states (generally negative) and motivations has been well-documented (Kim and Davis, 2009; Thatcher et al., 2008; Sun and Zhang, 2021). However, the understanding of the problematic AI chatbot use is far from conclusive. In particular, self-esteem is a strong predictor for different forms of problematic use, as evidenced by numerous studies examining social media, online games, and smartphones (Ahmed et al., 2021; Kardefelt-Winther, 2014b; Kim and Koh, 2018). Existing research posits that individuals with lower self-esteem are more likely to excessive engagement with social media and other IT products (Kircaburun et al., 2019). Therefore, we propose that users with low self-esteem are more susceptible to problematic chatbot use (PACU). Moreover, they often cope with their negative self-perceptions through two distinct pathways: direct confrontation or indirect avoidance, and this tendency leads them to rely more heavily on chatbots as a result of unmet social needs in the real world (Mehdizadeh, 2010; Ahmed et al., 2021). A recent study indicates that users with low self-esteem may evoke psychological states and motivations, they feel greater social anxiety and a higher tendency to escape from reality, which in turn significantly leads to problematic use intention (Liao et al., 2023; Gori et al., 2023). As such, we focus on low self-esteem individuals in the context of chatbot use, in which users are more likely to address their low self-esteem via social anxiety (direct confrontation) and escapism (indirect avoidance) while using chatbots.

This study addresses three gaps in the existing literature. First, we aim to examine how self-esteem influences users’ problematic behavior in the usage of AI chatbots. AI chatbot products can establish a substantial affective relationship with users that satisfy their needs of communication, exploration and entertainment (Chen et al., 2020; Skjuve et al., 2021), whereas social media refers to a virtual platform where users interact with others and online games provides opportunities for socializing and exploring the fictional world. Therefore, the influence mechanism driven by self-esteem may differ from previous studies. Second, this study draws on the compensatory internet use theory to explore how social anxiety and escapism mediate the influence of self-esteem on PACU. Cingel et al. (2022) implied that the association between self-esteem and IT use was complex and person-specific depending on their personal susceptibilities and usage patterns. Therefore, the process-based theory is necessary to understand the complex process of PACU in this study. Furthermore, unlike the previous literature, this study incorporates positive experience (i.e., AI chatbot flow) into the conceptual model besides negative psychological states and motivations (i.e., social anxiety, escapism) as mediators in order to reveal the dark side of flow experience. Therefore, this study constructs a multiple mediation model to investigate the complex process by which self-esteem produces PACU and reveal the importance of this independent variable as the formation mechanism. To our knowledge, this study provides a novel insight into under which conditions self-esteem would have a negative effect on PACU, and sheds more light on the importance of flow experience in the context of PACU. Additionally, the findings also have implications that may interest both users and practitioners in the context of AI chatbots.

## 2 Theoretical background and hypotheses development

### 2.1 Compensatory internet use theory

Rooted in research on internet addiction, Kardefelt-Winther (2014a) developed the theory of compensatory internet use to account for the possibility that excessive internet occurs, and gradually applied to problematic use and addiction to the internet and smartphones (Kardefelt-Winther, 2014b; Wang et al., 2015; Gori et al., 2023), which are considered as social stimulation. In the past few years, rapidly evolving AI-based internet products created a new virtual social environment for people that is different from the real world (Pentina et al., 2023). Internet users are attracted by chatbots and tend to indulge in the positive experience without caring about the true identity of their interlocutor (Adamopoulou and Moussiades, 2020), but this may also lead to over-reliance and addiction problems. In contrast to extant studies that highlight the compulsory and pathological aspects of internet addiction, the compensatory internet use theory posits that the perceived addiction phenomenon can be considered as a normative shift in the way of user’s entertainment or communication, and serves as evidence of the embeddedness of internet use into everyday life. Thus, addictive use and problematic use can be used interchangeably in the case (Tarafdar et al., 2020; Casale, 2020), defined as chatbot obsession, strong motivation and excessive time spent on using chatbots that impair people’s social activities, even health and wellbeing (Sun and Zhang, 2021).

Chatbot products are created to help people and facilitate their lives, which do not inherently cause negative consequences, rather, individual personality traits and what psychological problems have they been subjected to can affect whether chatbot use becomes problematic or addictive (Malik et al., 2023; Kardefelt-Winther, 2014b). Furthermore, the strong emotional connection that develops between humans and AI chatbots may lead to problematic use, which is associated with a persistent craving for companionship and enjoyment (Pentina et al., 2023). Drawing on the compensatory internet use theory, the problematic use of AI chatbots and the emergence of addictive behaviors can be understood as a form of compensation, whereby users excessively engage with AI chatbots to fulfill psychological and social needs that are unmet in their offline lives.

The theory assumes that interaction effects between the elements constitute the analytical focus rather than a direct relationship because singular psychological characteristics fail to be predictors of individual problematic use in the context of AI chatbots (Kardefelt-Winther, 2014a). The compensatory view frames that motivations play a key role in connecting psychological factors with negative outcomes (Kardefelt-Winther, 2014b). For example, low self-esteem might be associated with PACU, which was an indirect effect explained by the escapism motivation. Meanwhile, people with low self-esteem are more vulnerable to negative life situations, which may motivate them to use AI chatbots excessively to cope with dysphoric moods such as social anxiety (Kardefelt-Winther, 2014b; Hu et al., 2023). This offers an explanation for the PACU and its negative consequences without describing the behavior as pathological. Nevertheless, these relationships are yet to be explored for AI chatbots in problematic use. Moreover, people with negative self-traits also incline to develop positive experiences when using chatbots, leading to addiction or problematic usage tendencies of chatbots. This is what the theory of compensatory internet use has neglected.

### 2.2 Self-esteem and PACU

Self-esteem refers to a mental representation of oneself, and can be described as having respect for oneself, feeling positive about oneself, and accepting oneself based on the roles and areas of one’s life, indicating the overall sense of self-value (Rosenberg et al., 1989; Mann et al., 2004; Forest et al., 2023). People with high self-esteem have a positive self-image and feel confident in their abilities which are usually popular in online and real life, whereas low self-esteem person might focus more on their negative feelings and ignore the achievements they have (Zywica and Danowski, 2008; Forest et al., 2023). It is related to reflected appraisals, social comparisons, and self-attributions (Rosenberg et al., 1989).

The degree of self-esteem was found to have a significant negative effect on problematic IT product use and addiction, thereby making them vulnerable to losing hope for the real world which has been confirmed in the context of the internet, SNS, smartphones, and online games (Kim and Koh, 2018; You et al., 2019; Gori et al., 2023). Previous studies argue that people with low self-esteem are more likely to pass time using social media and internet apps for security and further form the excessive use habit, while people with higher self-esteem are active online but may not conduct addictive behavior because they have strong self-regulation ability and can protect themselves from negative experiences (Ahmed et al., 2021; Casale et al., 2022). Chatbots provides a more friendly social environment between human and AI. They always show their sense of humor and empathy while having interpersonal communication with users (Zhai and Wibowo, 2022). For people with low self-esteem, it is a compensatory platform. Based on the theory of compensatory internet use, individuals with low self-esteem often get into trouble in real-life situations, which prevents their emotional needs from being fulfilled. As a result, it increases the reliance on chatbots to seek emotional comfort as well as alleviate distressing feelings, and further produce PACU (Kardefelt-Winther, 2014c; Casale et al., 2022). Therefore, we hypothesized that:

H1: Self-esteem negatively influences PACU.

### 2.3 Social anxiety

Social anxiety refers to a psychological state and a set of physical symptoms of feeling scared and self-conscious of social situations where people anticipate excessive worries about being judged by others or whether their presence discomforts and bothers others (Jefferies and Ungar, 2020). This disorder impairs individuals’ ability to establish positive social relationships and intimacy, leading to a decline in overall mood and wellbeing (Sparrevohn and Rapee, 2009). Prior research has demonstrated that individuals with low self-esteem are more prone to experiencing social anxiety and perceive it as a direct confrontation (Kim and Davis, 2009). These individuals often view their future as bleak and hopeless, and they tend to respond negatively to uncertain situations (You et al., 2019).

The compensatory internet use theory suggests that social anxiety is positively associated with user’s problematic internet use (Kardefelt-Winther, 2014a; Kardefelt-Winther, 2014c). Similarly, individuals with social anxiety engage excessively in online communication as a compensatory mechanism to alleviate their anxiety during real-life social interactions (Fernandez et al., 2012). Consequently, individuals with a high degree of social anxiety exhibit an elevated risk of addiction to the internet and relevant applications (Yücens and Üzer, 2018; Zhang et al., 2019). Also, recent research has confirmed the effect of social anxiety on PACU (Hu et al., 2023). More importantly, the existing research has empirically examined that social anxiety significantly mediates the relationship between self-esteem and compulsive use of IT products (Kim and Koh, 2018). People with low self-esteem were found to be more inclined to seek out safe spaces to restore their sense of self-worth (You et al., 2019). Chatbots as anthropomorphic IT products, provide a convenient way for them to avoid real-life social situations by offering a sense of safety through pseudo-interpersonal contact and support with less stress and concern than actual social interaction (Hu et al., 2023). Users with low self-esteem may increase chatbot use frequency as a coping strategy when facing social anxiety so that they are easily addicted to chatbots as a safe haven to lean on. Therefore, we hypothesized that:

H2: Social anxiety significantly mediates the influence of self-esteem on PACU.

### 2.4 Escapism

The concept of escapism originally derives from “escape.” It represents a way of reality detachment that people use to divert their attention from unpleasant realities and distract themselves from daily pressures through imagination and immersion, indicating that the motivation of escapism is driven by one’s personal traits and social situation (Wu and Holsapple, 2014; Orazi et al., 2023). Self-esteem was sought as a predictor of escapism, and the impact of self-esteem was always found to be negative (Fernandes et al., 2021). Individuals with low self-esteem experience greater pressures and helplessness, they usually seek for an evasion shell where minimize their discomfort (De Jong et al., 2012). Thus, people with low self-esteem are more likely to use escapism as a coping mechanism for perceived self-deficiencies and devote themselves to the virtual world (Fraser et al., 2023).

Previous observations show that escapism is a common motivation among users who frequently use and engage in the internet and strongly induce problematic internet use (Gao et al., 2017; Miranda et al., 2023). For instance, Masur et al. (2014) and Smith (2022) posited escapism as one of the critical factors of social media addiction. Wang et al. (2015) showed that users are goal-directed when passing time with their phones and further confirmed the important role of escapism in problematic smartphone use. Hussain et al. (2021) considered addictive internet use as a negative outcome, which was potentially related to escapism. According to the theory of compensatory internet use, individuals who encounter negative consequences from using online gaming as a form of escapism aim to attempt to mitigate psychosocial problems or even symptoms through the internet and relevant IT products. Consequently, the relationship between escapism and internet addiction would be positive for users with low self-esteem (Kardefelt-Winther, 2014c).

Similarly, chatbots create a virtual world with human-like and emotion-driven interfaces to help users be away from sufferings, and myopic moods they have experienced in reality. Based on the view of psychodynamics, PACU could be defined as a psychological retreat that helps the addicted person cope with intense negative mental states, and such “anesthetic effect” happens in a context of self-regulation vulnerabilities such as low self-esteem which provides opportunities for forgetting about the troubles people encountered in real life (Khantzian, 1997; Di Blasi et al., 2020). Thus, we focus on the effect of self-esteem and escapism on PACU in which escapism could be thought as a mediating element.

H3: Escapism significantly mediates the influence of self-esteem on PACU.

### 2.5 AI chatbot flow

Flow indicates a psychological state of intrinsic enjoyment while individuals become totally absorbed in some motivated acts (Csikszentmihalyi, 1997). Flow theory emphasizes that flow refers to the optimal experience obtained from the acts and the compensation for individual motivation. Flow experience is verified to significantly influence users’ attitudes and behavioral intentions (Csikszentmihalyi, 1997). According to Hoffman and Novak (2009), flow exists in everywhere of our daily activities. It can be considered as a secret to happiness that helps people relieve daily stress even depression and has a positive impact on online activities engagement (Deng et al., 2010; Pelet et al., 2017). Therefore, internet companies strive to think about how to design their products (e.g., social media, online games) in a way that can enhance users’ flow experience.

AI chatbots are able to fulfill the needs of users as much as possible. They not only answer questions and solve problems related to work and study, but also master various conversation styles and respond emotionally to customers (Følstad et al., 2018), which arouses their curiosity and interest and further brings them flow experience. In other words, when engaging with chatbot conversations, users would have more positive flow experiences which leads to satisfaction and positive feelings. Despite the apparent advantages, flow may increase the frequency and duration of using chatbots. With time, flow states on chatbots may evolve into PACU (Kiss et al., 2020). Past studies suggested flow as a vital element for understanding the problematic use in the context of user’s online activities. For example, social media flow was reflected by ‘concentration,’ ‘time-distortion,’ ‘telepresence,’ ‘enjoyment,’ and ‘curiosity’ and was emphasized as an antecedent of problematic use behavior (Brailovskaia and Teichert, 2020).

More importantly, people with lower self-esteem are more likely to devote themselves to online entertainment where further forms reliance because they compensate for their difficulties in social relations while chatting with chatbots. Existing research has examined how users with low self-esteem experience stronger flow states through the use of the internet and smartphones (Khang et al., 2013). Although the compensatory internet use theory does not mention flow experience as its key element, it can be inferred that compared with negative events, individuals with low self-esteem tend to engage in addictive chatbot use after positive emotional states (Young et al., 2017). Moreover, a low level of self-esteem can incite a mentality of avoiding reality which may enable people to rest on activities with AI chatbots and intensify the compulsive use process. Miranda et al. (2023) confirmed that the flow state partially mediates the relationship between escapism and addiction. Therefore, given that people with low self-esteem easily have motives to escapism, this study posits that people experience AI chatbot flow while using AI chatbots, and such immersive experience subsequently drives them to PACU.

H4: Self-esteem negatively influences AI chatbot flow, which in turn positively influences PACU. In other words, AI chatbot flow significantly mediates the influence of self-esteem on PACU.

H5: Escapism and AI chatbot flow serially mediate the influence of self-esteem on PACU.

### 2.6 Research model

**Figure 1:**
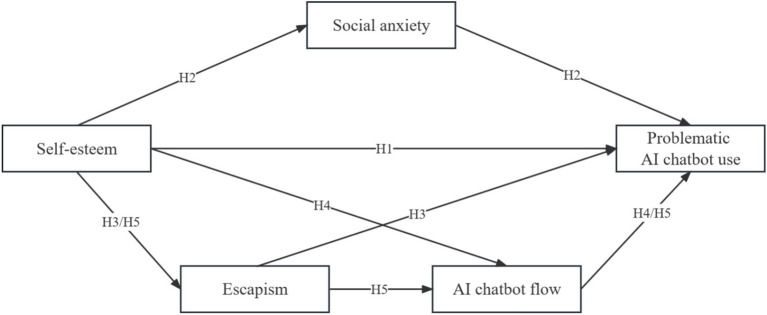
Conceptual model.

This study applies the theory of compensatory internet use as well as conceptualizes flow experience to discuss the influencing process by which self-esteem produces PACU. Then a conceptual multiple mediation model is proposed and this study explains how social anxiety, escapism and AI chatbot flow mediate the relationship between self-esteem and PACU. The conceptual model is demonstrated in Figure 1.

## 3 Methodology

### 3.1 Questionnaire development

We designed a questionnaire, including self-esteem as the independent variable, social anxiety, escapism and AI chatbot flow as mediators, and the dependent variable of problematic AI chatbot use (PACU). The questionnaire items must be developed because each construct cannot be directly observed. They were all adapted from guided by mature scales in previous studies and were measured on a 5-point Likert scale ranging from “1 = Totally disagree” to “5 = Totally agree.”

**Self-esteem** (Cronbach’s *α* = 0.935). The Rosenberg Self-esteem Scale (RSES) has been widely used to measure individuals’ self-esteem by asking the current feelings about themselves. This study used RSES as adapted into Chinese from previous studies of You et al. (2019) and Yang and Zhang (2022). The scale comprises eight items (e.g., On the whole, I am satisfied with myself.).

**Social anxiety** (Cronbach’s *α* = 0.913). The construct of social anxiety was adapted from Social Anxiety Scale for Social Media Users (SAS-SMU) which provides an overall evaluation of the level of individuals’ social anxiety (Alkis et al., 2017). The scale comprises seven items (e.g., I am afraid of interacting with others.). The scale validity was shown in an existing study conducted on Chinese online users (Zhang et al., 2019).

**Escapism** (Cronbach’s *α* = 0.817). The construct of escapism was adapted from Hirschman (1983) with four items (e.g., I think AI chatbots help me forget about troubles and pressures.). A recent study has shown the scale validity in the context of social media addiction (Miranda et al., 2023).

**AI chatbot flow** (Cronbach’s *α* = 0.877). This study adapted the scale of flow in human-computer interactions and the scale of SNS flow to measure the level of AI chatbot flow when using AI chatbots (Webster et al., 1993; Wang et al., 2017). The scale comprises six items (e.g., While using AI chatbots, I’m absorbed intently in what I was doing.).

**PACU** (Cronbach’s *α* = 0.877). The construct of PACU was adapted from the brief version of the Bergen Social Media Addiction Scale (BSMAS) (Andreassen et al., 2012) and Chinese Bergen Social Media Addiction Scale (Leung et al., 2020). The scale comprises five items (e.g., I tried to cut down on the use of AI chatbots but failed.) that assess the level of PACU. A recent study has shown the scale validity in the context of conversational AI addiction (Hu et al., 2023). It should be noted that item B3 of the BSMAS (‘Used social media to forget about personal problems’) was removed because AI chatbots are primarily used as communication tools and information providers, and their usage patterns are very different from those of social media platforms, which are more focused on immersive content consumption and social interaction. Removing this item helped make the scale more concise and focused on measuring problematic behaviors associated with the use of AI chatbots, while not affecting the analysis of escapist motivations.

**Control variables**. We measured five demographic variables as control variables that may influence the problematic use behavior toward AI chatbots (i.e., gender, age, education, occupation, and daily frequency of AI chatbot use). Each control variable was measured with single measurement items.

At the beginning of the questionnaire, we introduced the background and purpose of the study. The definition of chatbot and chatbot use were given to the participants as well as the confidentiality guarantees. A screening question was used to confirm that they have used at least one chatbot product. We then described distinct scenarios of chatbot use which is appended images so that participants can fully understand the content of the questionnaire. Furthermore, attention-checking items such as reverse wording and instructed response items were implemented throughout the questionnaire to check the participants who gave random answers (Shamon and Berning, 2020). Six experts were invited to review the content validity of the measures and modify the wording of the items. The expert group is composed of two professors in the field of information science, two internet practitioners, one psychology scholar and one linguistics expert. After the modification, a pre-test with small samples (*n* = 30) was performed. Each item was measured with the corrected item-total correlation above 0.3, and we ensured that the Cronbach alpha (*α*) exceeded 0.7. We then made the second modification based on their feedback. The complete list of measurement items appears in Appendix.

### 3.2 Data collection

The questionnaire was managed by a professional online survey platform.^1^ We promoted our need for respondents through social media and IT Q&A communities. We recruited participants based on the virtual snowball sampling method to increase sample size and representativeness, and participants were asked to spread the survey to their virtual social networks (Baltar and Brunet, 2012). The study was approved by the first author’s institutional Ethical Committee. All participants were fully informed about the study and agreed with the informed consent. In addition, we gave small monetary rewards to them after completing the survey.

At the time of the survey, the majority of the participants (70.2%) had completed university education. Nearly half of them (47.6%) were aged between 20 and 29 years old. Additionally, 40.1% of the sample consisted of employees. Notably, teenagers (individuals under the age of 18) were excluded from our sample owing to the ethical considerations that arise when conducting research involving adolescent populations.

The cross-sectional survey lasted days. We collected 643 completed questionnaires in total. Eighty unqualified responses were excluded owing to careless or dishonest responses. Finally, 563 questionnaires were collected with an effective rate of 87.56%. Table 1 summarizes the participant descriptive statistics.

**Table 1:** Characteristics of the participants.

Variable	*N*	%
Gender		
Male	306	54.35%
Female	257	45.65%
Total	563	100.00%
Age		
18–19	47	8.35%
20–29	268	47.60%
30–39	142	25.22%
40–49	88	15.63%
Above 50	18	3.20%
Total	563	100.00%
Education		
Junior high school or below	4	0.71%
High school/Vocational school	43	7.64%
College/University	395	70.16%
Master or above	121	21.49%
Total	563	100.00%
Occupation		
Student	177	31.44%
Enterprise employee	226	40.14%
Government	86	15.28%
Freelancer	69	12.26%
Unemployed/Others	5	0.89%
Total	563	100.00%
Daily frequency of AI chatbot use (hours)		
0.5 h or below	83	14.74%
0.5–1 h	130	23.09%
1–2 h	165	29.31%
2–5 h	121	21.49%
More than 5 h	64	11.37%
Total	563	100.00%

## 4 Results

### 4.1 Common method bias

Considering that the datasets were collected from online participants, the issue of common method bias (CMB) cannot be neglected where the online cross-sectional design is conducted. Therefore, this study corrected CMB problems through questionnaire remedies and post-statistical remedies. More specifically, clear instructions and items were given in our questionnaire. This study ensured the anonymity of responses in the survey process because reassuring the anonymity to the participants can reduce apprehensive evaluations and self-report bias (Podsakoff et al., 2012). After the survey was conducted, to check for the existence of CMB, we conducted Harman’s single factor analysis. The result of Harman’s one-factor test showed that a single factor explained 34.345% of the total variance, less than the 50% baseline. Additionally, based on the complete collinearity calculation, all the variance inflation factors (VIF) ranged from 1.557 to 2.977, which were below 5. Thus, common method bias is not a serious concern in this study.

### 4.2 Model estimation

The confirmatory factor analysis (CFA) was performed to assess the measurement model. The measurement model indicated a good model fit (*χ*^2^ = 1,000.457; df = 545; *χ*^2^/df = 1.836; IFI = 0.962; CFI = 0.962; TLI = 0.959; NFI = 0.920; NNFI = 0.959; RMSEA = 0.039; SRMR = 0.042). As shown in Tables 2, 3, the values of Cronbach’s *α* ranged from 0.831 to 0.935 while the composite reliability (CR) for each construct ranged from 0.838 to 0.936, and they all exceeded the threshold of 0.7, indicating satisfactory internal reliability (Hair et al., 2016). We ensured that the average variance extracted (AVE) values and the standardized factor loadings met the reference standard, indicating acceptable convergent validity (Hair et al., 2016; Shiau, 2023). Likewise, the discriminant validity was assessed by three criteria. Firstly, following the Fornell-Larker criterion, Table 4 presents that the diagonally presented square roots of the AVEs were above the corresponding pairwise off-diagonal correlations. Secondly, the HTMT score of each construct was lower than 0.85. Additionally, the average shared squared variance (ASV) and maximum shared squared variance (MSV) estimates did not exceed the AVE values, thus establishing discriminant validity (Table 5).

**Table 2:** Descriptive statistics for variables.

Construct	Mean	Median	SD	Min	Max
Self-esteem	2.988	3.125	0.994	1.000	5.000
Social anxiety	3.233	3.143	0.926	1.000	5.000
Escapism	3.391	3.500	0.889	1.500	5.000
AI chatbot flow	3.372	3.333	0.871	1.333	5.000
Problematic AI chatbot use	3.254	3.200	0.949	1.000	5.000

**Table 3:** Reliability and validity results.

Construct	Items	Loadings	AVE	MSV	ASV
Self-esteem	SE1	0.801	0.687	0.268	0.382
Cronbach’s *α*: 0.935	SE2	0.765			
CR: 0.936	SE3	0.802			
	SE4	0.823			
	SE5	0.780			
	SE6	0.792			
	SE7	0.812			
	SE8	0.839			
Social anxiety	SA1	0.783	0.657	0.268	0.250
Cronbach’s *α*: 0.913	SA2	0.727			
CR: 0.914	SA3	0.784			
	SA4	0.791			
	SA5	0.761			
	SA6	0.764			
	SA7	0.807			
Escapism	ES1	0.776	0.664	0.309	0.351
Cronbach’s *α*: 0.831	ES2	0.748			
CR: 0.838	ES3	0.793			
	ES4	0.658			
AI chatbot flow	ACF1	0.804	0.640	0.311	0.355
Cronbach’s *α*: 0.887	ACF2	0.792			
CR: 0.891	ACF3	0.722			
	ACF4	0.733			
	ACF5	0.696			
	ACF6	0.776			
Problematic AI chatbot use	PACU1	0.756	0.670	0.373	0.330
Cronbach’s *α*: 0.877	PACU2	0.768			
CR: 0.878	PACU3	0.808			
	PACU4	0.785			
	PACU5	0.717			

CR, Composite Reliability; AVE, Average Variance Extracted; MSV, Maximum of Shared Squared Variance; ASV, Average of Shared Squared Variance.

**Table 4:** The correlation matrix and square roots of AVEs.

	SE	SA	ES	ACF	PACU
SE	**0.829**				
SA	−0.485	**0.810**			
ES	−0.229	0.364	**0.815**		
ACF	−0.249	0.377	0.482	**0.800**	
PACU	−0.453	0.447	0.450	0.455	**0.818**

Square roots of AVEs (bold values) > latent variable correlation (LVC). SE, Self-esteem; SA, Social Anxiety; ES, Escapism; ACF, AI Chatbot Flow; PACU, Problematic AI Chatbot Use.

**Table 5:** The results of HTMT.

	SE	SA	ES	ACF	PACU
SE	–				
SA	−0.522	–			
ES	−0.253	0.416	–		
ACF	−0.271	0.414	0.559	–	
PACU	−0.501	0.499	0.523	0.509	–

SE, Self-esteem; SA, Social Anxiety; ES, Escapism; ACF, AI Chatbot Flow; PACU, Problematic AI Chatbot Use.

### 4.3 Endogeneity

Endogeneity problems are often caused by omitted variables that induce a correlation between the corresponding independent variables and the error term of dependent variables, which may further lead to erroneous conclusions. However, the issue of endogeneity has been largely ignored by research using PLS-SEM (Hult et al., 2018). Following suggestions noted by Hult et al. (2018) and Hair et al. (2019), the Gaussian Copula approach was used to detect the issue of endogeneity in this study. The results showed that neither of the Gaussian copula is significant indicating no potential endogeneity issue.

### 4.4 Structural model analysis

**Figure 2:**
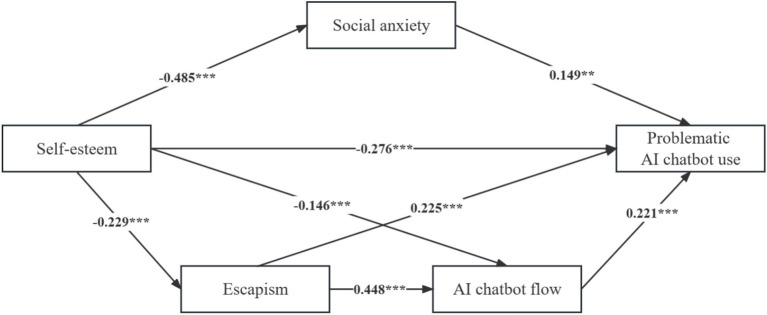
Structural model results.

This study implemented the Partial Least Squares-Structural Equation Modeling (PLS-SEM) by using SmartPLS 4.0, which was ideal for predictive modeling applications (Shiau, 2023). As shown in Figure 2 and Table 6, each the direct path coefficient ranged between −0.485 and 0.448, the t-statistics exceeded the 1.65 threshold, and all the paths were significant based on the *p* values (*p* < 0.05). More importantly, the direct path between self-esteem and PACU was significant (*β* = −0.276, *t* = 4.479, *p* < 0.001), thus supporting H1.

**Table 6:** Results of mediating effect test (bootstrap 5,000 samples).

Path/effect	Path coefficient	T statistics	*p* values	Conclusion
Direct effect				
SE → SA	−0.485	13.953	<0.001	***
SE → ES	−0.229	5.158	<0.001	***
SE → ACF	−0.146	3.886	<0.001	***
SE → PACU	−0.276	4.479	<0.001	***
SA → PACU	0.149	2.321	0.001	**
ES → ACF	0.448	12.396	<0.001	***
ES → PACU	0.225	3.318	<0.001	***
ACF → PACU	0.221	2.669	<0.001	***

Indirect effect	Effect	95% Boot CI	95% bias-corrected Boot CI	Type
SE → SA → PACU	−0.072	(−0.116, −0.032)	(−0.116, −0.032)	Complementary mediation
SE → ES → PACU	−0.052	(−0.081, −0.027)	(−0.083, −0.028)	Complementary mediation
SE → ACF → PACU	−0.032	(−0.054, −0.014)	(−0.056, −0.015)	Complementary mediation
SE → ES → ACF → PACU	−0.023	(−0.037, −0.011)	(−0.038, −0.012)	Complementary mediation

*p < 0.05. **p < 0.01. ***p < 0.001.

SE, Self-esteem; SA, Social Anxiety; ES, Escapism; ACF, AI Chatbot Flow; PACU, Problematic AI Chatbot Use.

The bootstrap procedure was performed to conduct the mediation analysis, which does not require samples with a normal distribution and is considered more suitable for testing the significance level of the indirect effects rather than the Sobel test. It was two-tailed whereas 5,000 samples bootstrapping procedure with a 95% confidence interval. The results of the mediation test support our hypotheses that there is a significant indirect effect in each relationship because zero is not included in both the 95% Confidence Interval (CI) and the 95% bias-corrected Boot CI (Zhao et al., 2010). According to Zhao et al. (2010), mediation was divided into complementary mediation, competitive mediation and indirect-only mediation, nonmediation was summed up into two patterns (i.e., direct-only nonmediation and no-effect nonmediation). The results showed that (1) social anxiety complementarily mediated the relationship between self-esteem and PACU (*β* = −0.072, SD = 0.021, 95% bias-corrected Boot CI = −0.116, −0.032); (2) escapism played a complementary mediating role in the relationship between self-esteem and PACU (*β* = −0.052, SD = 0.014, 95% bias-corrected Boot CI = −0.083, −0.028); (3) AI chatbot flow complementarily mediated the relationship between self-esteem and PACU (*β* = −0.032, SD = 0.010, 95% bias-corrected Boot CI = −0.056, −0.015); and specifically (4) escapism and AI chatbot flow serially complementarily mediated the relationship between self-esteem and PACU (*β* = −0.023, SD = 0.007, 95% bias-corrected Boot CI = −0.038, −0.012), which supported H2, H3, H4 and H5. As described by Zhao et al. (2010), complementary mediation appears when direct effect and indirect effect have the same sign, at this time, the total-effect test is considered superfluous.

Furthermore, Stone–Geisser Q^2^ and predictive power with prediction error assessment were calculated to examine the explanatory power of the model because they fit PLS-SEM well (Chin et al., 2020). Following the suggestion of Shmueli et al. (2019), the 10-fold and 10 repetitions prediction-oriented procedure was performed, and the Stone–Geisser Q^2^ and RMSE as metrics were assessed to confirm the predictive power. The Stone–Geisser Q^2^ for social anxiety, escapism, AI chatbot flow and PACU was 0.231, 0.047, 0.058 and 0.203 respectively, indicating medium, small, small and medium predictive relevance and accuracy of the PLS path model (Hair et al., 2019). Likewise, the indicators have lower PLS-SEM RMSE values compared to LM RMSE, indicating the satisfactory predictive power of the model (Shmueli et al., 2019; Chin et al., 2020).

### 4.5 Model comparison

Our aim was to identify the best predictive model among four competing models. As such, conducted a comparative analysis between our proposed model and three alternative models based on the extant psychological literature that finds distinct relationships among our latent variables (Yücens and Üzer, 2018; Iancu et al., 2015; Shen et al., 2024). Following Sharma et al. (2021) and Gudergan et al. (2025), we employed the Bayesian information criterion (BIC) and Akaike weights for model comparison. The criteria for selecting the optimal model were the lowest BIC and the highest Akaike weight. The findings revealed that Model 1 (our conceptual model) exhibited the most favorable BIC values and Akaike weights for the target variable PACU. Tables 7–9 summarize the results of the comparison between the conceptual model and the alternative models.

**Table 7:** Model 1 versus Model 2 for PACU.

Model	Path model	BIC	Akaike weights
Model 1	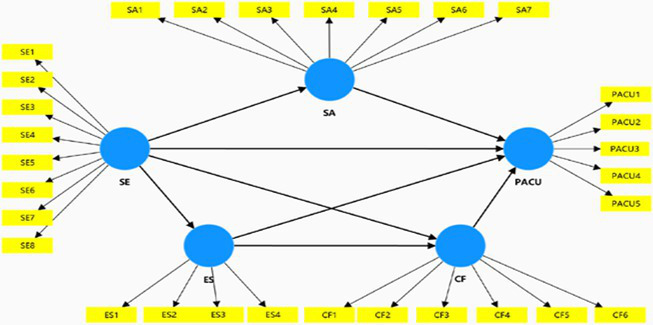	−250.700	0.960
Model 2	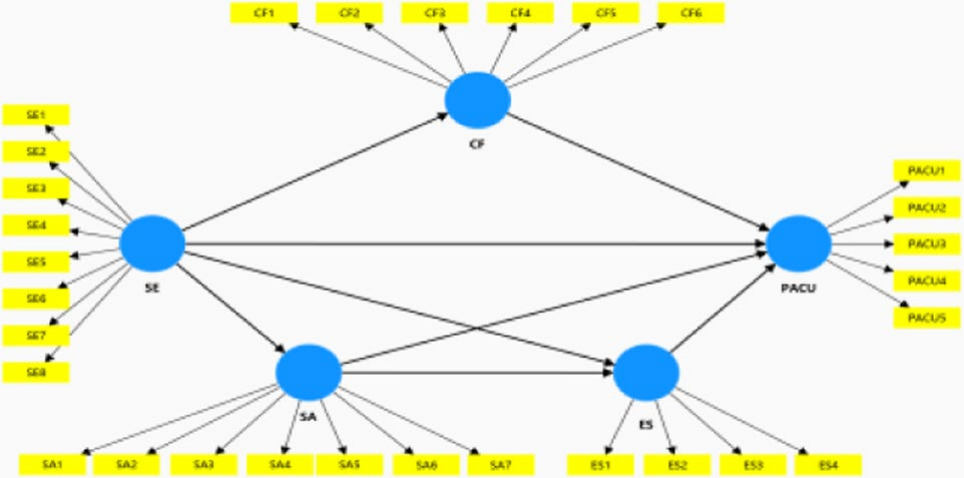	−244.327	0.040

**Table 8:** Model 1 versus Model 3 for PACU.

Model	Path model	BIC	Akaike weights
Model 1	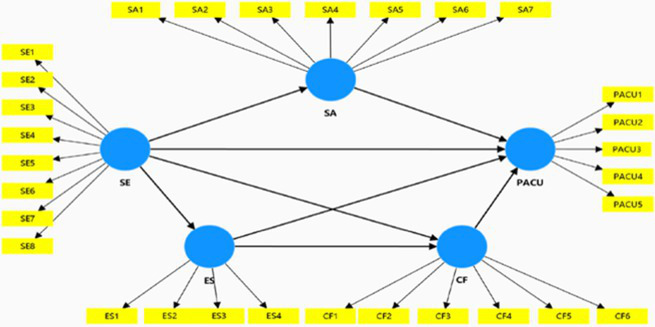	−250.700	1.000
Model 3	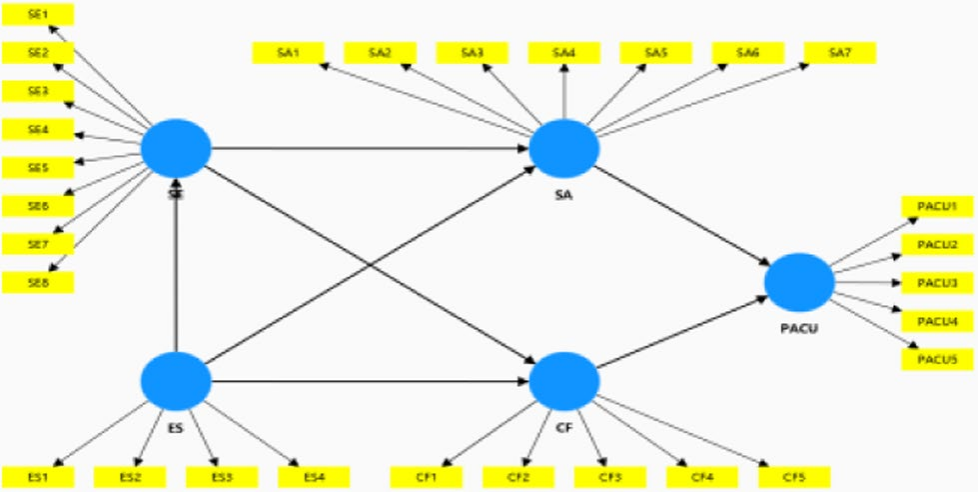	−180.072	0.000

**Table 9:** Model 1 versus Model 4 for PACU.

Model	Path model	BIC	Akaike weights
Model 1	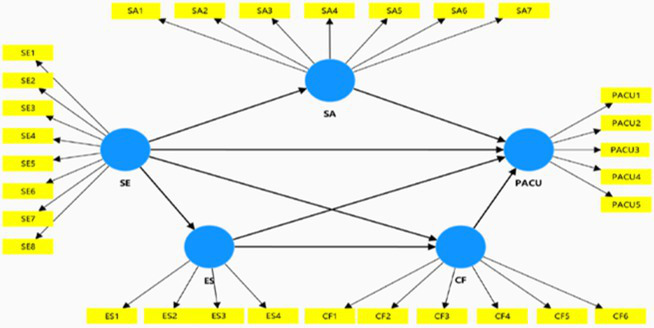	−250.700	1.000
Model 3	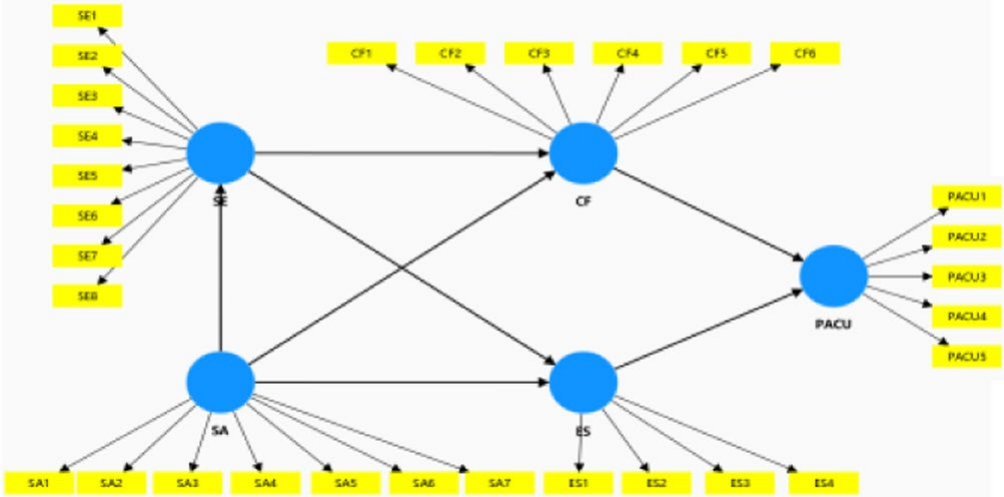	−171.324	0.000

## 5 Discussion

This study applied compensatory internet use theory to investigate the process by which self-esteem produces PACU, and explored the mediating roles of social anxiety, escapism and AI chatbot flow in this process. We developed a multiple mediation conceptual model which was done by collecting online cross-sectional data from 563 users who have interacted with at least one AI chatbot product. The empirical findings indicated that all the hypotheses were supported, thus revealing associations among factors in the conceptual model that we tested. Moreover, the path coefficient, statistical significance, t-statistics, and predictive power with prediction error assessment supported the conceptual model.

More specifically, the results demonstrated that self-esteem had a significantly negative impact on PACU, which is consistent with existing research about how self-esteem affects problematic and addictive use toward social media (Błachnio et al., 2016; Al-Samarraie et al., 2022). Those studies thought low self-esteem was the root of addiction. People with low self-esteem feel upset and frustrated when failing to cope with negative worries and problems, thus they begin to lose confidence in themselves and do not believe in their own self-worth (Guil et al., 2019; Yang and Zhang, 2022). Excessive chatting with AI chatbots such as Replika can downplay negative feelings and immerse them in positive feedback and encouragement, thereby enhancing their sense of self-worth (Jiang et al., 2022). According to compensatory internet use theory, PACU might be a dysfunctional strategy that allows people to compensate for the lack of self-acceptance and further get rid of negative feelings of low self-esteem by chatting with AI chatbots as external resources (Kardefelt-Winther, 2014a).

Social anxiety significantly mediates the negative effect of self-esteem on PACU. People with low self-esteem may feel nervous and out of control in interpersonal interactions due to their lack of social skills (You et al., 2019), so they are more likely to avoid talking with real people and turn to AI chatbots to fulfill their social needs. This supports the findings of Pentina et al. (2023) that users with the dominant social motivation tend to show greater reliance on AI chatbots. Moreover, people with low self-esteem may be vulnerable to the negative impacts of human relationships while having a high level of social anxiety. AI chatbots show intelligence and anthropomorphism, so that users can ask chatbots to change their language style, and they do not need to worry about saying the wrong thing or being rejected, which may increase users’ dependence on AI chatbots and gradually form a usage habit. Therefore, once they experience social anxiety, they are more likely to talk with AI chatbots due to the driving effect of the habit based on the habit theory of IT addiction (Wei et al., 2023).

Furthermore, escapism has a complementary mediating effect on the relationship between self-esteem and PACU. Users with low self-esteem who have a strong motive to escape reality tend to have a higher level of PACU than those who have high self-esteem. This is consistent with the argument of compensatory internet use theory on escapism and self-esteem (Kardefelt-Winther, 2014b) and with studies that highlight the negative effects of escapism on problematic IT use (Gao et al., 2017; Fraser et al., 2023). Escapism was studied as a hedonic gratification influencing continuance intention to use AI chatbots in the existing research (Mishra et al., 2022). It refers to a form of entertainment. However, in essence, escapism has both bright and dark sides which follows the criticism related to considering escapism as a unidimensional construct based on compensatory internet use theory (Kardefelt-Winther, 2015; Stenseng et al., 2023). This study points out that self-esteem exerts a negative effect on PACU via escapism because the dark sides of escapism would be exposed when people with low self-esteem fail to cope with troubles in real life, which in turn leads to negative and problematic outcomes.

The significant complementary mediating effect of AI chatbot flow on the relationship between self-esteem and PACU is a notable finding. Unlike highlighting the positive effect of flow experience in previous studies on AI chatbots (Baabdullah et al., 2022; Kautish and Khare, 2022), the findings of this study show that positive flow experience may cause maladaptive use behavior toward AI chatbots. The more intense the AI chatbot flow is, the higher PACU users with low self-esteem have. Self-esteem seems to be an important antecedent to experience AI chatbot flow. The findings reveal that the AI chatbot flow experience that low self-esteem evokes can contribute to connecting self-esteem and PACU. This supports Thatcher et al. (2008) who suggest that flow refers to the reason why users want to spend more time than intended engaged in an online activity, which leads to increases in problematic behavior. One possible explanation is that AI chatbots make users with low self-esteem immersive, and provide opportunities for perceived control that they hardly ever have in the real world.

More importantly, escapism and AI chatbot flow serially mediate the influence of self-esteem on PACU, suggesting that when people with low self-esteem hope to escape from their negative feelings or events they experience, they may focus on the interaction with AI chatbots and become overly dependent on AI chatbot as a maladaptive coping strategy. One possible explanation could be the perception of a lack of control when people with low self-esteem are driven by the need for escapism, thus occurring PACU. This finding also reveals that users who have a high level of escapism are also likely to be in a high flow state, and thus they might be experiencing time distortion and a loss of control over the long time on AI chatbots (Thatcher et al., 2008; Miranda et al., 2023). They might differ from those with low self-esteem who have higher flow producing higher PACU because of the effect of escapism. Escapism represents a motivation to unwind from constant self-consciousness by concentrating on specific activities that pull focus away from themselves (Baumeister, 1991). These activities may cause people to have flow which is a psychological state of ignoring time and outside distractions when using AI chatbots. Worse, if they rely excessively on flow for escapism while ignoring their environment in reality, they are at risk of developing PACU.

## 6 Conclusion

### 6.1 Theoretical implications

Although previous findings showed the possible adverse effects of the use of AI products (Coombs et al., 2021; Hu et al., 2023), few studies have focused on individuals’ problematic use toward AI chatbots and its underlying factors. This study investigated the complex process of problematic AI chatbot use (PACU) by examining the role of self-esteem as an influential antecedent. Given that the relationship between self-esteem and AI chatbots is complex and there exist multiple individual-level factors that govern this relationship, a multiple mediation model was proposed for the mediating roles that social anxiety, escapism and AI chatbot flow played in the effect of self-esteem on PACU. The findings indicate that users who have low self-esteem may develop a problematic outcome to the AI chatbot, either directly or through social anxiety or fulfilling their needs for escapism and/or through application-induced flow experience to AI chatbot services. The results of the multiple mediation model confirmed our expectations and built toward a more complete picture of the individuals’ psychological processes underlying PACU. The findings contribute to converting simple direct effects into multiple indirect effects and providing a novel insight of under which conditions self-esteem would have a negative effect on PACU.

This study constitutes the first attempt to adopt the compensatory internet use theory to reveal the psychological mechanism behind the maladaptive behavior toward AI chatbots, and further empirically verifies and extends the compensatory internet use theory in the context of AI technologies. Unlike the I-PACE model and Davis’ cognitive-behavioral model, which argue that pathological internet use is pathological, the compensatory internet use theory defines it as a coping strategy compensating for the needs that cannot be obtained in real life (Kardefelt-Winther, 2014a; Sun and Zhang, 2021). Thus, the assumption of the compensatory internet use theory is suitable for the common psychological states of online users. Furthermore, the findings verify the mediating role of social anxiety and escapism in the negative effect of self-esteem on PACU, which once again, highlighted the importance of activating both the motivation and psychological process for using AI chatbots to cause the problematic use behavior.

To extend the view of the compensatory internet use theory, this study integrates flow experience into the conceptual model to discuss the internal mechanism. In prior research, personal traits and negative psychological states as susceptibility risk factors affecting problematic internet behavior have always been in a critical position (Khang et al., 2013; Kircaburun et al., 2019; Ahmed et al., 2021; Sun and Zhang, 2021). Likewise, the compensatory internet use theory considers these elements as factors producing dysfunctional strategies. However, it should be noted that the positive experiences when using AI chatbots cannot be neglected besides self-traits and negative psychological states. The findings of this study found the mediation and serial mediating role of AI chatbot flow in the relationship between self-esteem and PACU. Flow represents the affective responses of online users, characterizing playfulness, and exploration as defining characteristics of human-computer interactions (Pelet et al., 2017). The findings reveal that even flow as a positive experience can lead to negative outcomes for people with low self-esteem because it provides opportunities for perceived control and allows them to ignore time and outside distractions. Therefore, this study sheds more light on the importance of flow experience in the context of PACU, and overturns the inherent cognitions.

### 6.2 Practical implications

The problematic or addictive use of AI chatbots is one of the factors of a controversial question about whether we can allow AI chatbots to do more for humans (Coombs et al., 2021). Although people are aware of the detrimental effects of problematic reliance on AI chatbots, a dilemma exists because it is hard for them to find a temporary relief method other than PACU but PACU poses a greater risk and cannot solve their negative feelings. For example, people with social anxiety feel less inhibited when they chat online with AI chatbots and further experience some compensation, but they still have problems initiating and maintaining offline relationships that they have to face (Stănculescu and Griffiths, 2022). Considering the findings of this paper, when the AI chatbot becomes a nuisance, practical implications can be provided for both online users and practitioners. From the perspective of AI chatbot users, understanding the underlying factors can help them increase their awareness of the potential risks associated with the use of AI chatbots. Likewise, understanding the mechanisms behind excessive and problematic use may help develop strategies to alleviate addiction problems. Therefore, the other practical implications of this study suggest that psychological and preventive interventions should address the specific mediating factors between self-esteem and PACU. Furthermore, educational awareness campaigns emphasizing the potential downsides of PACU can be implemented within social media, and virtual communities related to AI technologies. As for the practitioners in the AI chatbot industry, it is necessary to make healthy use AI announcements to guide users for the proper use of AI chatbots as well as develop the AI chatbot anti-indulged system, especially for adolescents. This will help users reduce problematic usage and promote AI technology for the better service of humankind.

### 6.3 Limitations

This study used first-hand cross-sectional data, which inherently limits our ability to investigate causal relationships. Furthermore, despite employing lie detection and instructed response items to mitigate careless or dishonest responses, self-reported questionnaires may still be subject to inaccuracies due to online users’ potential dishonesty regarding their experiences. Future research may consider adding experiments to improve its interpretation ability and provide a more reliable means of capturing users’ genuine responses. Second, while this study ensured the validity and reliability of the cross-sectional data, it is acknowledged that geographical and cultural factors may introduce specificity into the results, and problematic user behavior may vary across different regions of the world. In the following study, we will consider a multi-country comparison study on the problematic use of AI chatbots. In addition, the sample of our study may affect the generalizability of our findings. For example, adolescents may have unique behavioral patterns and vulnerabilities with technology, future studies are planned to conduct experiments and investigations specifically focused on teenager subjects.

## Data Availability

The raw data supporting the conclusions of this article will be made available by the authors without undue reservation.

## Footnotes

^1^ https://www.wjx.cn/

## Appendix: Constructs and measurement items

**Table:**

Construct	Items	Sources
Self-esteem	SE1. On the whole, I am satisfied with myself.	You et al. (2019) and Yang and Zhang (2022)
	SE2. At times I think I am no good at all. (Reverse-scored)	
	SE3. I think I have a lot of good qualities	
	SE4. I think I can do as well as most people	
	SE5. I feel I do not have much to be proud of. (Reverse-scored)	
	SE6. I certainly feel useless at times. (Reverse-scored)	
	SE7. All in all, I am inclined to feel that I am a failure. (Reverse-scored)	
	SE8. I take a positive attitude toward myself.	
Social anxiety	SA1. I am afraid of interacting with others.	Alkis et al. (2017) and Zhang et al. (2019)
	SA2. I feel anxious about not being able to meet people’s expectations.	
	SA3. I feel nervous when I have to talk with others about myself.	
	SA4. I feel anxious about the fact that others might find my actions awkward.	
	SA5. I feel uneasy while making new friends.	
	SA6. I feel tense when I meet someone for the first time.	
	SA7. I feel anxious when talking with people I have just met.	
Escapism	ES1. I think AI chatbots help me forget about troubles and pressures.	Hirschman (1983)
	ES2. I think AI chatbot helps me escape from the world of reality.	
	ES3. AI chatbots help me avoid loneliness.	
	ES4. AI chatbots help me get what I want without much effort.	
AI chatbot flow	CF1. While using AI chatbots, I’m absorbed intently in what I am doing.	Webster et al. (1993) and Wang et al. (2017)
	CF2. Using AI chatbots provides me with a lot of fun.	
	CF3. Using AI chatbots bores me. (Reverse-scored)	
	CF4. Using AI chatbots excites my curiosity.	
	CF5. Using AI chatbots often makes me forget where I am.	
	CF6. Time flies when I am using AI chatbots.	
Problematic AI chatbot use	PACU1. I spent a lot of time thinking about AI chatbots or planned use of AI chatbots.	Andreassen et al. (2012) and Leung et al. (2020)
	PACU2. I felt an urge to use AI chatbots more and more.	
	PACU3. I tried to cut down on the use of AI chatbots but failed.	
	PACU4. I become restless or troubled if I have been prohibited from using AI chatbots.	
	PACU5. I used AI chatbots so much that it has had a negative impact on my job/studies.	
